# Use of the Dexamethasone-Corticotrophin Releasing Hormone Test to Assess Hypothalamic-Pituitary-Adrenal Axis Function in Rheumatoid Arthritis

**DOI:** 10.1155/2009/391284

**Published:** 2009-09-29

**Authors:** Eman A. Hasan, David S. Jessop, Lynsey L. Power, Paul T. Monk, John R. Kirwan

**Affiliations:** ^1^The Academic Rheumatology Unit, University of Bristol, UK; ^2^Welcome Laboratories for Integrative Neuroscience and Endocrinology, University of Bristol, UK

## Abstract

*Objectives*. Hypothalamic-Pituitary-Adrenal axis function may be abnormal in rheumatoid arthritis (RA). A pilot study in 7 patients suggested impaired glucocorticoid feedback in some patients after the dexamethasone-corticotrophin releasing hormone (CRH) test. This study aimed to investigate the dexamethasone-corticotrophin releasing factor test in a larger group of patients and relate the results to characteristics of the disease. *Methods*. Outpatients with active RA (≥3 swollen and tender joints and C-reactive protein > 10 mg/L) took dexamethasone (1.5 mg) at 23:00 hour in the evening. Next day, baseline saliva and plasma samples were collected, CRH was infused at 11:00 hour, and 4 serial blood and saliva samples were collected. Plasma samples were stored at −80°C and a radioimmunoassay performed for saliva and plasma cortisol. *Results*. All 20 participants showed normal dexamethasone suppression and mounted no response to the CRH challenge. In samples with measurable cortisol, there was a strong correlation between saliva and plasma values (*r* = 0.876, *n* = 26, *P* < .01). *Conclusion*. No abnormalities were found in the Dexamethasone-CRH test in RA patients in contrast to a previous pilot study. Salivary cortisol measurement may offer an alternative noninvasive technique to plasma cortisol in RA patients in future studies.

## 1. Introduction

Rheumatoid arthritis (RA) is a multifactorial chronic inflammatory joint disease that involves secretion of proinflammatory cytokines such as tumour necrosis factor-*α* (TNF-*α*), interleukin 1 (IL-1), and IL-6 which are associated with local inflammation and a systemic reaction [1, 2]. The hypothalamic-pituitary-adrenal (HPA) axis and the systemic sympathetic and adreno-medullary (sympathetic) system are the major physiological pathways which mediate responses to stress, controlled centrally from the hypothalamus and the brain stem, whose main function is to maintain basal and stress-related homeostasis [3, 4]. Thus a feedback system operates through the CNS, the HPA axis and the hypothalamic-autonomic nervous system (HANS) [5] Cytokines induce increased release of adreno-corticotrophic hormone (ACTH) (and hence cortisol) [6, 7] through production of corticotrophin releasing hormone (CRH) and arginine vasopressin (AVP) [4, 8]. CRH and AVP exert a synergistic action on the release of ACTH, and cortisol exerts a negative feedback action on ACTH and CRH [9]. Because of the importance of cortisol as an anti-inflammatory compound, HPA integrity and cortisol production in RA have been extensively investigated. No major defects in the HPA axis have been reported in RA but subtle abnormalities have been identified and it is generally accepted that the HPA axis response to inflammation in RA is inadequate [10]. 

 The corticotrophin releasing hormone (CRH) test was developed as a technique for assessing HPA axis integrity, and its further more sensitive modification, the dexamethasone-corticotrophin-releasing hormone test (Dex-CRH test), has been used to investigate changes in HPA axis function in patients with depression [11, 12]. In the Dex-CRH test the pituitary release of ACTH in response to CRH infusion is prevented by the prior administration of dexamethasone, a commonly used therapeutic corticosteroid. The subsequent administration of exogenous CRH provides a measure of the sensitivity of the corticotropes to CRH which in turn reflects the degree of synergy with endogenous AVP release into the portal blood. Studies in depressed patients have noted an increased cortisol response to CRH following dexamethasone, suggesting decreased central feedback sensitivity to circulating corticosteroids. 

 We have previously reported a pilot study using the Dex-CRH test to investigate possible abnormalities in HPA axis activity in RA patients, and found that 3 of 7 patients failed to suppress their cortisol response to CRH after taking dexamethasone [13]. We did not investigate any underlying mechanism for this phenomenon but speculated that it may be a consequence of impaired glucocorticoid feedback in this subgroup of patients, a phenomenon which may be due to downregulation of glucocorticoid receptors (GRs) [14] or GR polymorphisms [15–17]. Our initial investigation was limited to a small number of patients with very active disease. While suggesting that some patients have disruption in HPA control, it was not possible in this limited study to relate this to past or future disease activity. Therefore, the present study was undertaken to include a larger number of patients with a range of disease activity, severity, duration, and age, and with the intention of comparing the subsequent course of disease in those with and without abnormal Dex-CRH responses. On the basis of the pilot study, we anticipated that the present study would reveal a larger number of RA patients with an early cortisol release from dexamethasone. We hypothesised that this subgroup of patients would display increased disease activity when clinically assessed at 6 and 12 month time points after the study. 

 As an additional investigation, we also measured salivary cortisol in RA patients. Saliva sampling has been used as a non-invasive technique to measure unbound bioactive levels of cortisol in normal healthy subjects, and in a wide range of psychological and pathophysiological conditions [18, 19]. Subtle dysfunctions in basal and stress-induced cortisol secretion have been reported in RA patients [10, 20] and salivary sampling would be advantageous for use in future investigations of HPA axis activity in RA. A strong positive correlation between blood and salivary cortisol has been reported in healthy subjects [20, 21]. However, many patients with RA have secondary Sjogren's syndrome, which causes reduced and altered saliva secretion. This may impair the ability of salivary cortisol concentrations to adequately reflect plasma concentrations. Therefore we investigated the correlation of plasma and salivary cortisol in RA patients.

## 2. Materials and Methods

### 2.1. Patients

Outpatients with active RA (≥3 swollen and tender joints and C-reactive protein (CRP) > 10 mg/L) were recruited to the study. Participants were selected to be widely representative in terms of age, gender, disease severity, and duration. Patients that had received glucocorticoid therapy within 6 weeks prior to the study were excluded, as were patients on anti-TNF therapy, premenopausal women, and those taking heparin, vasopressin, or undergoing renal dialysis. Almost all the patients were taking nonsteroidal anti-inflammatory drugs. Transport and refreshments were provided. We did not incorporate a healthy control group into the study design since in our previous study [13]. there was such a large and clearcut difference between patient suppressors and nonsuppressors that we felt justified in predicting a similar outcome this time. Therefore we predicted that we would identify two quite distinct patient groups, the subgroup of patients who demonstrated an early escape from dexamethasone suppression by mounting a cortisol response to CRH, and the patients whose cortisol was suppressed following the DEX-CRH test. This suppressor group would act as the control group. Given this study design, and the question it addressed, we considered it unnecessary, and therefore unethical, to include a control group of non-RA patients. 

### 2.2. Ethics

Ethical approval was obtained from the Research Ethics Committee of United Bristol Healthcare NHS Trust and all participants gave written informed consent.

### 2.3. Procedure

In addition to their normal medications, patients were given 1.5 mg dexamethasone to take orally at 23:00 hour on the eve of the study. Patients were given a reminder telephone call approximately one hour before that time. The following morning, patients were admitted to the Rheumatology Day Case Unit at 09:30 hour where an intravenous cannula was inserted, usually in the antecubital fossa, and flushed with 5 mL 0.9% saline. This flush was repeated after each blood sample aspiration, and the first 3 mL of each aspiration was discarded to avoid dilution effects. Baseline blood and salivary samples were taken at 10:00 and at 10:30 hour. Blood samples were placed immediately on ice, and quickly transferred to EDTA tubes for centrifuge, after which plasma was promptly separated, aliquoted, and stored on dry ice until transfer to a −20°C freezer. Salivary samples were obtained by asking the patients to dribble into specimen tubes. The samples were stored at −20°C. At 11:00, 100 *μ*g human CRH (ClinAlpha, Merck Chemical Limited, Nottingham, England) reconstituted in 5 mL 0.02% HCl in 0.9% saline was infused through the cannula over 30 seconds. Further blood and salivary samples were taken at 11:30, 12:00, 12:30 and 13:00 hour. Patients remained seated throughout the procedure. Blood pressure (BP) and heart rate (HR) were recorded prior to and post-CRH administration as well as adverse reaction during or post-CRH infusion. The mean ± standard error of mean (SEM) for pre- and post-CRH systolic BP was 144 ± 3.9 and 131 ± 3.3 mm/hr and the pre- and post-CRH diastolic BP were 82.5 ± 2.5 and 81.6 ± 2.5 mm/hr, respectively. The pre- and post-CRH HR were 76.9 ± 1.5 and 82.1 ± 1.8 beats/min, respectively. 

### 2.4. Clinical Assessment

On the day of the study, participants were asked to assess the extent to which they were affected by pain, fatigue, and their disease in general using 10 cm visual analogue scales (VASs), disability using the Health Assessment Questionnaire (HAQ) score, and disease activity using the Disease Activity Score 28 (DAS28). A full clinical history was taken, including details of disease severity and duration, and details of all medications and other illnesses (particularly those known to influence HPA axis regulation). Standard clinical assessments of RA were carried out, including swollen and tender 28 joint counts and clinician's overall assessment of disease using VAS and DAS28. Blood tests for the acute phase response, C-reactive protein (CRP) and plasma viscosity (PV), were taken if not already obtained within the previous week. X-rays of the hands were taken if not done within the previous 6 months.

### 2.5. Laboratory Measurements

CRP and PV analyses were performed by the routine hospital laboratory. Salivary and plasma cortisol were measured by radioimmunoassay in sodium citrate/sodium orthophosphate buffer at pH 3. Saliva samples were diluted in buffer and assayed in duplicate with radiolabelled iodine 125-cortisol and antiserum. Total assay tube volume was 0.3 mL [22]. Cortisol antiserum is a rabbit polyclonal antibody raised against cortisol-3-BSA (B391, Acris Antibodies, Hiddenhausen, Germany). Cross-reactivities are prednisolone 36%, 11-deoxycortisol 10%, corticosterone 3.2%, and cortisone 0.9%. Inter- and intraassay coefficients of variation are both less than 10%. After 24 hours incubation at 4°C, activated charcoal was added to each tube and tubes were centrifuged for 15 minutes. Supernatants were discarded and radioactivity in the pellets was measured on a gamma counter [23]. 

### 2.6. Statistics

Sample size was initially set at 40 patients, to allow for discrimination in outcomes between groups of normal and abnormal Dex-CRH responders which, on the basis of the pilot study, were expected to be about 50% each. Analysis was planned for after the first 20 patients had been included to confirm the anticipated ratios. Descriptive statistics (mean and 95% confidence intervals) were calculated for patient characteristics and for plasma cortisol at each time interval. Each CRH response was classified as “responder” or “nonresponder” according to the pattern observed and without knowledge of patient or disease characteristics. Responders would be expected to have cortisol increases greater than those shown by normal volunteers and similar to the three “responder” patients in our previous report [13]. 

## 3. Results

**Figure 1:**
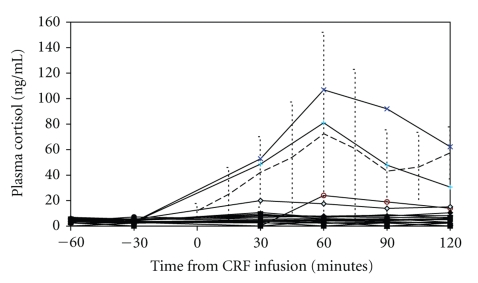
Dex-CRH Test in 20 patients. Each line shows the cortisol values for one patient. The dotted line shows the mean and 95% confidence interval for normal subjects previously reported [13].

The clinical characteristics and results for the first 20 patients included in the study are shown in Table 1. A list of medication taken by the patients recruited is provided in Table 2. Baseline plasma cortisol after oral dexamethasone and the cortisol responses to CRH for each patient are shown in Figure 1. Also included in Figure 1 are the mean and 95% confidence intervals (CIs) for normal controls as previously reported [13]. Following dexamethasone administration the previous evening, baseline plasma cortisol concentrations were low in all the participants. There was no abnormal cortisol response to CRH challenge in any patient. Although 3 subjects did mount a small cortisol response each was within the range previously observed for healthy control subjects, and neither approached the response expected without dexamethasone suppression (>200 ng/mL).

**Figure 2:**
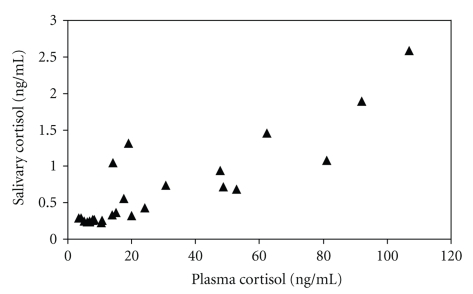
Plasma and Salivary Cortisol. Cortisol values for simultaneous plasma and salivary samples where the value for salivary cortisol was above the limit of detection.

In those salivary samples (*n* = 26) which had cortisol concentrations above the detection limit of the assay (0.2 ng) there was a positive correlation between plasma and saliva cortisol (*r* = 0.876, *P* < .01) (Figure 2).

## 4. Discussion

All 20 patients with RA showed a normal dexamethasone suppression of cortisol at baseline and mounted no response to the CRH challenge in contrast to the previous pilot study, in which a subgroup of RA patients failed to show cortisol suppression. Although 3 patients in the present study did mount a small cortisol response each was within the normal range, therefore we are unable to show further evidence for a subgroup of RA patients with an abnormal HPA axis response to the Dex-CRH test. We considered several possible reasons for the discrepancy. Firstly, the seven patients included in the previous pilot study were inpatients admitted to the hospital because of a flare of their RA. Now we infrequently admit patients with severely active RA (since the era of antitumour necrosis factor and other forms of biologic therapy) and our participants were outpatients. However, they showed a wide range of disease activities with mean DAS28 score of 4.23, and some patients had higher disease activity than the pilot study patients. Patients on current anti-TNF therapy were excluded, so avoiding previously reported alterations in steroid metabolism [24, 25]. Secondly we considered the time at which the study had been conducted. In the present study, the test was performed in the morning while in the pilot study it was performed in the afternoon. However the half life of dexamethasone is greater than 36 hours, therefore patients in both studies should have been well suppressed by the dexamethasone at the time of the CRH infusion. 

We also considered if the CRH may have been inactive. The majority of our patients experienced “hot flushes”—a mild, short-term sensation of warmth felt in the head, neck, and upper part of the body which is a well recognised effect of CRH infusion, showing that our preparation was bioactive, also blood pressure decreased and heart rate increased immediately after CRH. Finally we considered the possibility that 3 of the 7 patients in the previous study did not take their dexamethasone tablets the night of the study. In the present study, we telephoned each patient the night before the test to remind them about their tablets. Our results do not exclude HPA axis dysregulation, but further investigation in RA may require the measurement of a wide range of hormonal and immune responses including cytokine levels following stressor challenge. 

Although we found that no patients mounted a cortisol response to CRH greater than the 95% confidence interval for normal healthy volunteers in our previous study [13], it did appear that 18 out of 20 patients had lower levels than the normal subjects in response to CRH infusion, although due to slight differences in the protocols between studies it was not appropriate to compare the two sets of data to determine statistical significance. While some studies have reported abnormal cortisol responses to the CRH test in RA patients, the majority have not (reviewed in [10, 26]). In the present study we addressed the question whether different subsets of RA patients can be distinguished by the Dex-CRH test and we did not design the study to examine differences in responses to CRH between patients and healthy controls. Therefore any such differences, if they exist, must be the subject of a larger investigation with a different study design incorporating healthy participants as the controls.

Measurement of salivary cortisol is a common method to assess HPA axis activity in RA [27–30] but to our knowledge, this is the first paper that has studied the correlation between salivary and plasma cortisol in RA patients. Plasma and salivary cortisol have been measured in a study testing cortisol elimination from plasma in premenopausal women with RA, where the elimination profiles were similar, but correlation analysis was not reported [29]. However, some RA patients have hyposalivation and reduced buffering capacity [31–33] and 25–35% of RA patients have secondary Sjogren's syndrome [34, 35], which might alter the relationship between plasma and salivary concentrations. We have found a strong correlation (*r* = 0.876) between saliva and plasma cortisol concentrations in those patients who have detectable salivary cortisol. Therefore, salivary cortisol can be used as an alternative to plasma cortisol in RA patients, offering a convenient, reliable, and non-invasive method for cortisol measurement. If developed as a suitable home-use kit it might avoid hospital attendance for sample collection in studies involving RA patients.

## 5. Conclusion

In conclusion, in contrast to an initial pilot study, we found no abnormalities in the Dex-CRH test in the 20 patients with RA. These negative results are important as they are evidence supporting HPA-axis integrity in RA. In addition, a strong correlation between saliva and plasma cortisol concentrations in RA suggests that salivary measurements may offer an alternative to plasma cortisol in future studies. 

## Figures and Tables

**Table 1:** Characteristics of patients*.

Age (years)	63.1 (10.5)
M:F	6:14
RA duration (years)	14.7 (10.8)
Tender joint count	18.4 (8.9)
Swollen joint count	7.6 (5.3)
CRP (mg/L)	25.5 (26.7)
Patients with erosions (%)	12 (60)
Patients with RF (%)	14 (70)
Pain (VAS, 0–100 mm)	51.8 (22.0)
Patient overall severity (VAS, 0–100 mm)	49.3 (21.6)
Fatigue(VAS, 0–100 mm)	62.2 (23.5)
Health assessment questionnaire score	1.894 (0.454)
Disease activity score (DAS28)	5.7 (1.2)

*Mean and SD (standard deviation) unless indicated,

RA: rheumatoid arthritis, CRP: C-reactive protein,

VAS: visual analogue scale.

**Table 2:** Medications of the participants.

Treatment	No. of patients (%)
NSAIDs	10 (50)
COX-II inhibitors	2 (10)
Methotrexate (MTX)	10 (50)
Sulphasalazine (SSZ)	2 (10)
MTX and SSZ	5 (40)
Gold	1 (5)

NSAIDs: Nonsteroidal anti-inflammatory drugs,

COX-II: cyclo-oxygenase II.
